# Specific considerations for research on the effectiveness of multisectoral collaboration: methods and lessons from 12 country case studies

**DOI:** 10.1186/s12992-021-00664-w

**Published:** 2021-02-01

**Authors:** Rachael Hinton, Corinne Armstrong, Eriana Asri, Klaus Baesel, Sarah Barnett, Carla Blauvelt, Saidatul Norbaya Bt Buang, Louise Bury, Jai K. Das, Jennifer Franz-Vasdeki, Helia Molina Milman, John Murray, Susana Palma, Ilona Renner, Marion Roche, Victoria Saint, Sarah Simpson, Lucy Singh, Diana Vaca McGhie, Daria Ukhova, Jetske van Dijk, Silvia Xinico, Helga Fogstad, Wendy Graham, Shyama Kuruvilla

**Affiliations:** 1 Rachael Hinton Editing, Geneva, Switzerland; 2 Independent consultant, London, UK; 3 Nutrition International, Jakarta, Indonesia; 4 Deutsche Gesellschaft für Internationale Zusammenarbeit (GIZ) GmbH, Bonn, Germany; 5 SB Consultancy World, Bristol, UK; 6 VillageReach, Lilongwe, Malawi; 7 Family Health Development Division, Ministry of Health, Kuala Lumpur, Malaysia; 8 Global Research Consultancy, Ipswich, UK; 9 Division of Women and Child Health, Aga Khan University, Karachi, Pakistan; 10 Independent consultant, Seattle, USA; 11 University of Santiago, Santiago, Chile; 12 Independent consultant, Iowa City, Iowa USA; 13 USAID Health Education and Policy Project, Guatemala City, Guatemala; 14 National Centre for Early Prevention, Federal Centre for Health Education, Koeln, Germany; 15 Nutrition International, Ottawa, Canada; 16 Bielefeld University, Bielefeld, Germany; 17 EquiACT, Lyon, France; 18 EGA Institute for Women’s Health, University College London, London, UK; 19 American Heart Association, Washington, DC USA; 20 Independent consultant, Glasgow, UK; 21 Cherie Blair Foundation, (Formerly Child to Child), London, UK; 22 Alianza Nacional de Organizaciones de Mujeres Indigenas por la Salud Reproductiva Nutrición y Educación (ALIANMISAR), Guatemala City, Guatemala; 23 Partnership for Maternal, Newborn & Child Health, Geneva, Switzerland; 24 London School of Hygiene and Tropical Medicine, London, UK; 25 World Health Organization, Geneva, Switzerland

**Keywords:** Multisectoral collaboration, Sustainable development goals, Research, women’s health, Adolescent health, children’s health

## Abstract

**Background:**

The success of the Sustainable Development Goals (SDGs) is predicated on multisectoral collaboration (MSC), and the COVID-19 pandemic makes it more urgent to learn how this can be done better. Complex challenges facing countries, such as COVID-19, cut across health, education, environment, financial and other sectors. Addressing these challenges requires the range of responsible sectors and intersecting services – across health, education, social and financial protection, economic development, law enforcement, among others – transform the way they work together towards shared goals. While the necessity of MSC is recognized, research is needed to understand how sectors collaborate, inform how to do so more efficiently, effectively and equitably, and ascertain similarities and differences across contexts. To answer these questions and inform practice, research to strengthen the evidence-base on MSC is critical.

**Methods:**

This paper draws on a 12-country study series on MSC for health and sustainable development, in the context of the health and rights of women, children and adolescents. It is written by core members of the research coordination and country teams. Issues were analyzed during the study period through ‘real-time’ discussions and structured reporting, as well as through literature reviews and retrospective feedback and analysis at the end of the study.

**Results:**

We identify four considerations that are unique to MSC research which will be of interest to other researchers, in the context of COVID-19 and beyond: 1) use theoretical frameworks to frame research questions as relevant to all sectors and to facilitate theoretical generalizability and evolution; 2) specifically incorporate sectoral analysis into MSC research methods; 3) develop a core set of research questions, using mixed methods and contextual adaptations as needed, with agreement on criteria for research rigor; and 4) identify shared indicators of success and failure across sectors to assess MSCs.

**Conclusion:**

In responding to COVID-19 it is evident that effective MSC is an urgent priority. It enables partners from diverse sectors to effectively convene to do more together than alone. Our findings have practical relevance for achieving this objective and contribute to the growing literature on partnerships and collaboration. We must seize the opportunity here to identify remaining knowledge gaps on how diverse sectors can work together efficiently and effectively in different settings to accelerate progress towards achieving shared goals.

## Introduction

The success of the Sustainable Development Goals (SDGs) is predicated on multisectoral collaboration (MSC), and the COVID-19 pandemic makes it ever more urgent to learn how this can be done better. Complex challenges facing countries, such as COVID-19, cut across health, education, environment, financial and other sectors. Addressing these challenges requires the range of responsible sectors and intersecting services – across health, education, social and financial protection, economic development, law enforcement, among others – transform the way they work together towards shared goals.

Existing challenges and inequalities experienced by women, children and adolescents, are and will continue to be exacerbated by COVID-19 and require a multisectoral response [1]. The downstream effects of public health distancing measures, such as shutting down schools and non-essential businesses, quarantine and social isolation measures and avoiding large crowds, affect the poor and vulnerable the most and have a disproportionate effect on women, children and adolescents [2]. For example, lockdowns and quarantine due to COVID-19 contributed to reported rises in domestic violence, requiring multiple sectors to work together to ensure appropriate care and support for survivors of violence [3]. During COVID-19 restrictions health and social support services need to be reinforced and extended, such as emergency phones and 24-h hotlines and temporary shelters for survivors. First responders must also be adequately equipped to address violence against women and make prompt referrals to support services. The security and justice sectors need to promptly process complaints and protection orders and adjust security restrictions during the pandemic, such as in Spain where women who leave a situation of violence are exempt from lockdown. Partnerships with communication and private sector providers can help to expand technology-based solutions. One example is smart phone applications which can be used during lockdown restrictions to increase access to information on violence against women, service provision, and data collection [4, 5].

The need for MSC is not only vital in the context of the COVID-19 pandemic. The SDGs are explicitly multisectoral and require a joined up way of working to tackle interconnected global health, environmental, social, economic, and institutional challenges [6]. SDG 17 places partnerships and cooperation at the centre of sustainable development efforts. The need for establishing multi-stakeholder partnerships for sustainable development is explicitly encouraged at the SDG target level. Against this backdrop MSC is actively promoted as a central mechanism for the realization of the SDGs. Well before the SDGs, several important initiatives and policy frameworks considered partnerships as an effective instrument for realizing health and sustainable development, beginning with the 1978 Alma Ata Declaration on Primary Health Care [7–10].

We use “multisectoral collaboration” to mean multiple sectors and stakeholders intentionally coming together and collaborating in a managed process (i.e. not ad hoc) to achieve shared outcomes and common goals [11]. We used this definition for a series of multi-country studies conducted in 2018 by 12 low-, middle- and high-income country teams to identify “what works” in MSC at the intersection of health and sustainable development [12]. Others have used a similar definition to allow for any combination of organizational types such as public–private partnerships, public–non-government partnerships, and whole-of-government initiatives working in specific policy and topical areas, including those relevant to the 17 SDGs [13–15]. Multisector collaboration, cross-sectoral action and intersectoral action refer to a similar process and are often used synonymously.

While the necessity of MSC is recognized, research is needed to understand how sectors collaborate, inform how they could do so more efficiently, effectively and equitably, and ascertain similarities and differences across contexts. How it should be done in practice, or what works and does not work is not always clear both under conditions of an immediate crisis response and for sustainable development. To answer these key questions, research to strengthen the evidence-base on MSC is critical. Recent publications have looked at country case studies on how MSC works for health and sustainable development [11], the governance of multisectoral action [16], MSC research priorities on MSC for health and sustainable development [13, 17] and methodological gaps in studies of MSC [14]. To address these methodological gaps it is suggested more attention be given to the use of conceptual frameworks and mixed-methods, the organizational arrangements for collaboration, engaging non-traditional stakeholders, and the MSC context [14]. However, these points appear to be more about research best practice overall than about what is unique to research on MSC.

This commentary builds on previous work to identify four considerations that are unique to MSC research and which will be of interest to other researchers undertaking studies of MSC. We draw primarily on the 2018 series of multi-country studies, with details on the 12 MSCs, actions taken and outcomes published elsewhere [12]. In the discussion we reflect on the relevance of these considerations in the context of the COVID-19 pandemic and beyond.

## Methods

This commentary is written by members of the 12-country study series research coordination and country teams. Issues were analyzed during the study period through ‘real-time’ discussions and structured reporting, as well as through literature reviews and retrospective feedback and analysis at the end of the study. We were able to reflect on what happened and why during the study, identify strengths and adaptations made, as well as areas for improvement.

For this paper we draw on four sources of information. First, the 12 country teams leading the development of each case study and seven international consultants provided ‘real time’ feedback during study coordination meetings and communication via email and phone calls. Second, towards the end of the study, eleven of the 12 country teams completed an online evaluation survey. The survey included both Likert scale and open answer questions about country teams’ experiences and learnings from the research process. A reporting template was also completed by six of the seven international consultants who provided technical and writing support to the country teams. Although time constraints prevented all the country teams and international consultants from completing the survey and reporting template, all had previously given ‘real time’ feedback. Lastly, retrospective post-study discussions were held by the country case study teams, the coordination team and partners during the launch of the series at the Partnership for Maternal, Newborn & Child Health (PMNCH) Partners’ Forum in New Delhi, India and subsequently by the authors in developing this paper. Three authors (RH, LS, JFV) analyzed the qualitative data and quantitative survey responses. Through discussions they identified emerging themes and key findings which were reviewed by co-authors.

## Specific considerations for research on MSC

### 1. Use theoretical frameworks to frame research questions as relevant to all sectors and to facilitate theoretical generalizability and evolution

Both qualitative and quantitative methods are required to study the unique contexts and considerations of MSCs. Therefore, research based on a theoretical framework is important as generalizability would largely be based on theoretical generalizability, that is recurring concepts and themes across contexts, rather than statistical sampling generalizability [18]. Regarding theoretical frameworks, there is a body of literature on partnership approaches towards achieving the SDGs, including on joined-up strategies across government, whole of government approaches, Health in All Policies (HiAP), and interorganizational collaborations [19–23]. This literature covers a range of issues from describing key determinants of success and MSC outcomes to governance, implementation and coordination considerations. However, there was limited explanation of what works in practice for MSCs, which was the focus of the 12-country study series.

A literature review was conducted to examine how action across sectors was planned, implemented, and sustained at national or subnational levels [24]. It included a draft conceptual framework to explain the “how to” of collaborating across sectors rather than merely the “what” of collaboration or the “importance” of collaboration. The literature review highlighted key considerations for what works in practice for MSCs. These considerations included identifying and defining a problem requiring MSC, forming the MSC, planning, budgeting and implementing, developing shared norms and communication, evaluating success and sustaining collaboration across sectors. However, no one model covered the full range of issues highlighted in the literature review that were specific to MSCs.

**Fig. 1:**
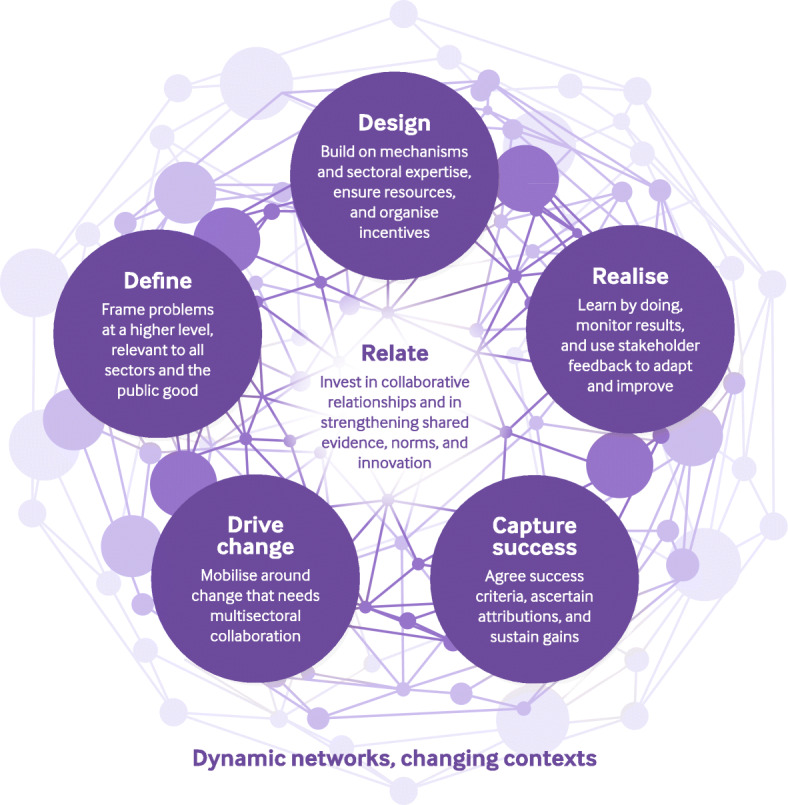
A multisectoral collaboration model to achieve transformative change

Since no MSC theoretical model was available that covered all these issues, we decided to use a multi-grounded theory approach [25], starting with a policy science model that was well aligned with all the considerations highlighted in the MSC literature review [26]. Being based on an overview of policy science, political philosophy and public administration literature, this model theoretically also applied across all sectors. The objective was that this model could then be adapted and evolved through the case study findings to reflect MSC-specific considerations. The model was used to frame the research questions, and was then tested, adapted and customized as a theoretical framework specifically for MSC based on the findings from the multi-country studies (Fig. 1) [11].

For example, with reference to the define element of the model and applying that to identifying and defining the problem requiring MSC, a case study from South Africa studied the “She Conquers” multisectoral campaign which aimed to reduce the burden of HIV among women aged 15–24 years. For the MSC, this problem was defined not as a biomedical issue of disease transmission or of reducing viral loads, but in terms of girls’ and young women’s agency and empowerment [12]. This broader framing was important so different sectors could see the relevance of the problem to their work, which facilitated their engagement and collaboration.

We adapted the model to highlight and emphasize points specific to MSCs. We added the component of ‘capture success’ to address the case study findings that markers of MSC success can be measured across a spectrum of results and need to be defined in a way that all sectors agree. This is further elaborated in Point 4. We also modified the model with the ‘relate’ component to highlight the sustained investment needed in collaborative relationships and the mechanisms to strengthen them [11].

Since we began our study, other publications have highlighted similar components for research on MSCs, including for developing a framework on multisector and multilevel collaboration in South Africa [15], strategies for the governance of MSCs for health in low-and middle-income countries [16] and to identify methodological gaps in the study of MSCs [14] (Table 1).

**Table 1:** Emerging alignment of multisector collaboration (MSC) theoretical and conceptual frameworks/ models, illustrative examples

Multisectoral collaboration model on transformative change from multi-country studies series (Kuruvilla et al. 2018)	Framework on multisector and multilevel collaboration for HIV/AIDs governance in South Africa (Mahlangu et al. 2019)	Strategies to govern multisectoral collaborations (Rasanathan et al. 2018)	Domains related to identifying methodological gaps for MSC research (Glandon et al. 2019)
**Dynamic networks, changing contexts:** The collaboration occurs within wider interactions and networks and changing political, social, and environmental context	Pre-conditions: buy-in to the process; recognized interdependencies; resources; and prior relations (history of interaction)	Understand the key actors and political ecosystem, including type of MSC required and mapping incentives, interests and hierarchies	Contextualisation: key contextual factors affecting MSC likelihood, formation, implementation, impact, etc. across place, time, topic, partner type(s), etc., including nature and extent of their influence on MSCs
**Drive change:** Set agendas and mobilize a critical mass of stakeholders for change; ascertain whether the situation is best tackled by MSC; and optimise linkages across sectors and SDGs			Initiation: Key opportunities, conditions or drivers for MSC formation; appropriate scope and scale; which partners to engage and when and how to engage them
**Define:** define the problem in a way that improves how solutions are assessed, and enables stakeholders to agree on a course of action and develop a well-defined project	Key drivers / requirements: shared understanding of the problem and common goal; strategic planning; leadership; and capacity	Frame the issue in the most strategic manner; define clear roles with specific sets of interventions according to sector	
**Design:** build on existing mechanisms and sectoral expertise to plan programmes; set up governance for the MSC; and develop solutions and innovations that are relevant to stakeholders, contexts, and goals	Mechanisms and processes: set up mechanisms for interaction, communication, conflict management and building trust. Structure: definition of membership and expectations, roles and responsibilities, and operating procedures	Use existing structures unless there is a compelling reason not to do so	MSC governance structures and attributes: leadership; voice, inclusiveness and representation; roles and responsibilities; accountability and information sharing mechanisms
**Realise:** strengthen implementation, monitoring, and evaluation as iterative and adaptive processes that facilitate learning from successes and failures; and adapt to change	Execution: implementation of the plan; coordination of activities; and constant reflection and learning	Develop financing and monitoring systems to encourage collaboration; strengthen implementation processes and capacity	MSC implementation: key strategies, approaches, challenges and success factors; building capacity for engagement; maintaining stakeholder commitment
**Relate:** systematically assess and strengthen synergies between sectors; manage MSCs; and promote multistakeholder dialogue and deliberation	Administration: setting up meeting and sending out invites; documentation of engagements; and following up action plans	Pay explicit attention to the roles of non-state sectors; address conflicts of interest and manage trade-offs; distribute leadership	Adaptation: key factors and actions affecting sustainability of MSCs over time; adapting MSCs to changing conditions; whether, when and how to conclude MSCs
**Capture success:** use a range of qualitative and quantitative methods to monitor and evaluate results comprehensively and promote learning from both successes and failures; and formulate MSC as an intervention to which health and development outcomes can be attributed	Evaluation: measuring the outcome of collaboration; accounting for multisectoral action	Support mutual learning and implementation research	Measurement: indicators or assessments of MSC inputs/costs, functioning, outputs, outcomes and/or impacts; value-add of MSC vs single-sector approaches; attributing results to MSC components or partners

The alignment of our MSC model with emerging MSC theoretical and conceptual frameworks in different contexts further strengthens the theoretical generalizability.

### 2. Specifically incorporate sectoral analysis into MSC research methods

Highly complex problems, such as achieving sustainable development, are better solved by networks of diverse actors interacting and collaborating both inside and outside of government. The 2030 Agenda calls for multistakeholder partnerships to share knowledge, expertise, technology and financial resources for achieving the SDGs globally. This form of collaborative participation can make solutions more effective [27, 28]. However, in line with identified strategies for governing MSCs more specifically [16], stakeholders must first assess whether collaboration across sectors is a better way to achieve desired changes than reliance on action by an individual sector. When a decision to engage in MSC is taken, incorporating a sectoral analysis into the research to understand how different sectors are identified, characterized and interact as a collaborative network is critical [29]. MSCs differ in the number of sectors and stakeholders involved and the breadth of scope can range from pilot programmes to those at scale. MSCs also occur within changing political, social and environmental contexts, so that different sectors may be more or less strongly engaged at different stages in the collaboration.

A sectoral analysis would also examine negative (“trade-offs”) and positive (“co-benefits” and “synergies”) interactions across the collaborative network, especially during the development of specific solutions [29]. This would give important insight into issues of authority and leadership for the MSC and why different sectors judge it worthwhile to work together and in what ways, as opposed to other possible alternatives e.g. each sector working separately [30]. Such insights can inform the planning, design and implementation of MSCs in order to manage or avoid such anticipated challenges.

Research could also consider the innovation and incentives for sectors to work together and the collaborative arrangements, both formal and informal, that support the MSC. For example, in the case study series we found establishing a formal cross cutting coordination function, through, for example, ministries of planning or finance, was shown to be helpful for connecting diverse technical sectors, deciding budgets, and for engaging a wide range of government and non-government stakeholders for a common purpose [11]. MSCs also commonly set up formal mechanisms for timely, open communication among multisectoral stakeholders including the public. In other cases, informal brokering and networking is used to build relationships and trust across sectors [11, 12, 27].

### 3. Develop a core set of research questions, using mixed methods and contextual adaptations as needed, with agreement on criteria for research rigor

Based on the components in our model we developed ashared indicators of success and failure core set of questioshared indicators of success and failurens to assess how MSC’s work. We produced key factors to consider for each question, which needed to be adapted and refined for each collaboration context (Table 2). For example, in the case study series we found that the extent to which an MSC was formalized within an institutional structure varied greatly. We also found questions on resource allocation or sustainability were difficult to apply consistently. In Malawi the Chipatala Cha Pa Foni (CCPF) (Health Center by Phone) developed from an innovative idea by an NGO and other stakeholders, into a nationwide government-owned collaboration, with hotline staff now funded by government [12]. This can be compared with the case study in Guatemala where the programme was funded by short term-grants from donors, and the collaboration was dependent on the unpaid work of Indigenous female volunteers [12]. In response, researchers will need to adapt the key factors to ensure relevance to their contexts and capacities.

**Table 2:** Examples of core research questions for research on MSC

Why is an MSC needed in this instance?	
● factors driving change; actors driving change; identifying sectors to be involved; policy context within which change is being considered	
Why is the problem relevant for different sectors?	
● nature of the problem requiring MSC; sector interests, incentives and trade-offs; co-benefits and creating a shared vision	
How is the MSC designed to address the problem?	
● mobilising resources (financial, human, organizational); funding allocation and cost-sharing; operational structures/mechanisms; accountability structures; beneficiary and stakeholder engagement; data and information sharing	
How is the MSC implemented?	
● process of monitoring, evaluation and learning; role of different sectors; challenges and adaptations; piloting; sustainability; institutionalisation	
How are relationships maintained across sectors?	
● role of champions; formal and informal trust building; stakeholder and community engagement	
What are markers of success (and failure)?	
● assessing results, sectoral gains and attributing impact; adaptiveness; scale up; enabling/challenging factors; lessons learned; stakeholder perceptions of the MSC	

A mix of qualitative and quantitative methods is required to answer the questions in Table 2. The methods used in the multi-country study included review of literature and context; identification and collation of existing quantitative and qualitative data; key informant interviews and other new data collection as needed; multistakeholder dialogues; and analysis and synthesis of findings [31]. The time and resource commitments corresponded with a similar multistakeholder process conducted in ten low-and middle-income countries in 2014 [32] however, the 2018 series of studies raises pecific considerations for research on MSC.

For example, it is necessary to clearly identify and agree on which sector is leading the MSC, including who has authority for convening a multistakeholder dialogue across sectors. This can be complicated in some contexts when the sector which plays a coordinating and administrative function (e.g. health) is different to the sector(s) which oversees the budget (e.g. finance) or is responsible for service delivery (e.g. education, transportation, water and sanitation, defence and security, public administration etc). The importance of incorporating a sectoral analysis (Point 2 above) into the research is therefore critical for answering the core research questions. Concerns related to confidentiality, data protection, and information sharing across sectors and administrative levels can also be a challenge for the MSC itself as well as for research on MSC.

In multi-country studies there is often diversity and divergence with research teams in disciplinary background and in how research quality across qualitative and quantitative methods is understood. Setting out clear research quality criteria (Table 3) can help to develop shared understanding across disciplines. It can also encourage theoretical generalizability through the application of other theoretical frameworks in research to evolve knowledge for MSC, including theories of partnership working [34, 35], collaborative networks [36] and interorganisational collaboration [37, 38]. Research quality criteria can also help ensure relevance of the research questions, rigour in the research methods used and the interpretation of results.

**Table 3:** Criteria to ensure rigour in quantitative and qualitative research [33]

Quality criteria	Quantitative	Qualitative
Generalizability	- Statistical generalizability	- Analytical/theoretical generalizability; transferability within and across contexts
Validity	- Accuracy of measurement - Validity: face, construct, criterion	- Appropriateness of methods and expertise and experience of researchers - Validity: democratic (all perspectives accurately represented); dialogic (review and deliberation of findings); process (cogent and dependable); outcome (resolution of research question)
Reliability	- Precision - Replicability: inter-observer, test-retest, triangulation	- Auditability and transparent documentation of methods - Consistency in applying methods - Achieving theoretical saturation
Credibility	- Triangulation of data sources - Counterfactual analysis and causal inference	- Triangulation of data sources - Expertise and experience of researchers - Diverse perspectives to test and refine the findings, including consideration of alternative interpretations
Context for application of quality criteria	- Embedded in a broader understanding of and expertise in quantitative research design, data analysis, application, and limitations	- Embedded in a broader understanding of and expertise in qualitative research design, data analysis, application, and limitations - In-depth understanding of context of analysis from different stakeholder perspectives and ‘thick description’

### 4. Identify shared indicators of success and failure across sectors to assess MSCs

Just as the research question could be framed differently so that it is relevant to all sectors, researchers must look at different measures of progress, beyond outcomes or impact related to single sector or discipline. This would also align with the globally agreed results framework of the SDGs. We found MSCs define progress in many different ways ranging from process measures to health and development outcomes [11]. Such measures should not only be relevant for all sectors, but also appropriate to the context and timespan of the MSC itself. The breadth of scope of the MSCs in the multi-country study series varied from an MSC that was established for a finite period to accomplish a specific goal, to a sub-national programme, to an MSC at national scale. Therefore, researchers must be open to a broad spectrum of how to define progress within strategies and programmes. For example, country teams had to overcome familiarity with reporting on standard health and development indicators to also collect data on qualitative and process outcomes such as the strength of collaborative relationships at different levels. Looking at different kinds of impact will also require the harmonisation of monitoring and evaluation systems and the sharing of data across sectors, with joint responsibility for, and ownership of, results.

To further advance understanding of the context of MSC, understanding the factors that enable MSCs to flourish or conversely to not work effectively together is critical. Significant lessons can be learned from approaches that do not work or problems encountered as well as successful adaptations to challenges and contexts. Although the research considerations in Table 2 encouraged critical reflection and descriptions in terms of ‘what did not work’, many country teams in the 2018 study series were reluctant to report on challenges and failures, as the case studies were viewed as success stories. Countries in the study series were selected from a global call for proposals for evidence of “success” in MSC [39]. This had implications for the ways in which MSC outcomes were captured and the analysis subsequently written up.

A fear of negative findings is not however specific to MSCs and framing the research as an opportunity to learn and improve is essential. For example, the Indonesia country team recognised their most important programmatic learning was due to the identification and understanding of challenges. They found because the MSC was designed by one sector and implemented by another, programme goals and motivation were not always aligned across sectors [12]. This learning approach also reflected a major finding from the series which shows regular monitoring and evaluation is a valuable part of the MSC for programme implementation and course-correction to achieve desired results [11].

## Applying lessons for studying multisectoral collaboration in the context of COVID-19

The significant social, economic and political implications of the coronavirus pandemic demonstrate the interconnectedness of the SDGs and reminds us that building a robust evidence base on how to achieve effective MSC is an urgent priority. This paper contributes new knowledge on research methods for studying MSCs. To build the evidence, we propose four specific research considerations for future research on MSC.

Firstly, the knowledge base on MSC research, while limited, consistently highlights the overarching principle that one size does not fit all. Multisectoral collaborations are inherently context specific, complex and heterogenous [11]. We therefore took a pragmatic approach to the development of the theoretical model for the case study series. We also propose a series of tools to support its application [11]. The findings highlight the political nature of partnerships and collaboration, such as how MSCs are framed, coordinated, resourced and measured. We briefly reference the growing literature and other frameworks related to these issues, such as on network governance, interorganisational collaboration, joined- up government and HiAP, and as illustrated in Table 1. We encourage future research to further develop theoretical frameworks and engage with this literature on collaboration and partnerships and address the political dimensions of these efforts.

Because MSCs are forged in response to a unique problem or opportunity, this also determines the stakeholders and sectors involved [11, 40]. In the context of COVID-19, an inclusive framing for MSC research would enable all sectors – health, water and sanitation, education, as well as cross-cutting areas such as gender, human rights, planning and finance among others – to see the relevance of the research, understand their respective roles and where they could best contribute their sectoral expertise in a coherent, connected way. These are important reasons to ensure that a research question and objectives are framed in a way to be relevant to all sectors.

Secondly, MSC is a dynamic process and stakeholders and their engagement may change across different components and contexts of the collaboration. MSCs also utilise a range of mechanisms and structures to support their collective action across sectors. We recommend future studies of MSC undertake a sectoral analysis to better understand these diverse ways of working, the structures that support or potentially discourage MSC, and different sectors’ contribution and actions. Assessing the co-benefits of interaction across sectors and the potential trade-offs can help to understand issues of authority and how conflict is managed as well as illuminate common interests and unexpected alliances through the MSC process [29, 30]. These may also reflect the synergies and trade-offs between the 17 SDGs and 169 targets.

In response to COVID-19, many governments have set up multisectoral task forces to bring together sectors that are typically siloed. UN organizations such as the World Health Organization have also developed essential resources and good practices for coordinating a multisectoral response to COVID-19. A sectoral analysis is therefore critically important for research on MSCs in the context of COVID-19 to understand both the informal and formal mechanisms for collaboration, including if and how existing structures were built on, and if the MSC is robust and relevant enough to be useful moving forward. Exploring stakeholder assessments about the need to collaborate, or at least coordinate both during and beyond the pandemic is central to this analysis. In such a crisis, the situation is more complicated due to the speed of action required, the number of sectors and stakeholders involved and the decisions to make around the division of labor and funds to reach people during the pandemic. To maximize positive interactions for the MSC and mitigate negative ones [30] it would be important to identify the ‘boundary spanning’ stakeholders who facilitate the sharing of information, build common understanding and manage relationships during the pandemic [41].

Thirdly, we recommend a range of methods from diverse disciplines be employed to study MSC. A series of core questions should be considered in light of different theoretical frameworks and adapted to different contexts. A combination of qualitative and quantitative methods is also needed to produce the type of evidence and knowledge that can really inform decision-making. This point is exemplified in the context of COVID-19 as it would be necessary to consider key questions related to how approaches to MSC may have changed, during and beyond the pandemic. For example, the implications of restrictions on large gatherings may have contributed to novel and innovative forms of communication and mechanisms for collaboration across sectors and with the public. Democratic transparency and open information, supported by the technological deployment of public-health measures has been shown to be critical for citizen engagement in the COVID-19 response [42].

Because the scope and nature of studies of MSC is diverse and contextual changes might occur, research questions and methods must be flexible and adaptable throughout the process to ensure the relevance of the findings. Understanding rigour in mixed-methods research is critical for multidisciplinary research teams. For future studies, researchers could benefit from practical guidance for establishing and assessing rigour for an integrated mixed-methods study of MSC, beyond addressing quantitative and qualitative quality criteria. These issues are especially critical for MSC research in the COVID-19 context where qualitative and quantitative data is being harnessed in multiple ways across relatively short timescales, including to predict and measure the spread of disease, track and monitor behaviour, coordinate volunteers, and identify the most vulnerable.

Lastly, the SDGs provide a shared framework with agreed health and sustainable development indicators beyond a single sector or discipline and which give consideration to cross cutting issues such as human rights and gender equality [29]. Definitions of success in MSCs also go beyond standard health and development metrics and include the process and dynamics of the collaboration itself, for example on the dynamics and strength of relationships across sectors [12, 29, 40].

This is particularly relevant in the context of COVID-19 given social distancing and lockdown interventions aimed at reducing COVID-19 transmission impact on other sectors such as the economy, education, safety and security, as well as other areas of health, such as mental health, domestic violence and treatment seeking and access to care for other health conditions. Therefore, MSC research impact could range from stemming the spread of the disease, to community knowledge and practice (e.g. of new social distancing norms), to measures of human rights and civic engagement. The strength and sustainability of the MSC itself should also be considered, such as measures of awareness and understanding among stakeholders about the need for effective MSC, during and beyond the pandemic.

This could help to help transform a study from reporting on indicators to exploring the “why” and “how” of related changes in the context of the MSC and that are translatable into action. Understanding what works and what does not work in MSC, especially during times of crisis, should also be analysed from multiple perspectives. It could also provide useful lessons on how to capture and report on common priorities across sectors for women, children and adolescents to inform future actions.

In attempting to understand and communicate the success factors of MSC, important contextual factors and stakeholder relationships which influence what does not work may be missed. Challenges and failures provide opportunities to improve ways of working and has longer term implications for the design, implementation and impact of MSCs, as emphasized in the editorial accompanying multi-country series [12]. Areas for further work must focus on the development and standardisation of indicators, including measures of success and failure. The framing of studies explicitly around challenges as well as successes would encourage a genuine “learning society” approach. The MSC series shows that countries are willing to use these methods and generate new learning when the process is made inclusive and stakeholders can see the benefits of working together.

Researchers need to strike a balance between measures of progress and methodological processes that are as robust as possible from a research perspective but also feasible, timely and useful for capturing the reality of MSC and to inform decision making for policy and practice. The unprecedented nature of COVID-19 provides a valuable opportunity to design and test innovative research MSC approaches to track different measures of progress to inform the short and long-term response to the pandemic and its reporting. In doing so it would also be important to understand if the hard-learnt lessons of the multisectoral response to the Ebola epidemic have been taken on-board to avoid repeating the same mistakes [43]. Similarly, to prevent entrenching social, health and environmental inequalities that followed the 2008 financial crisis, there are calls to ensure the response to the economic recession stimulated by the pandemic looks to the good of the whole of society, and especially women who make up the majority of the newly unemployed, and not just focus on the economy [44, 45].

## Conclusion

How MSCs influence health and sustainable development, including in situations of crisis, is a dynamic and evolving research area. With this paper we offer a unique contribution to building the evidence base on how to study MSC. Sharing and learning lessons about how diverse stakeholders in different sectors do more together efficiently and effectively in different settings is vital for effective response to the COVID-19 pandemic, as well as to accelerate progress towards achieving the SDGs by 2030.

## Data Availability

The datasets used and/or analysed during the current study are available from the corresponding author on reasonable request.
